# Prognosis of concomitant users of clopidogrel and proton-pump inhibitors in a high-risk population for upper gastrointestinal bleeding

**DOI:** 10.1186/2050-6511-15-22

**Published:** 2014-04-15

**Authors:** Qing Wang, Rickard Ljung, Jesper Lagergren, Yunxia Lu

**Affiliations:** 1 Department of Molecular Medicine and Surgery, Karolinska Institutet, SE-171 76 Stockholm, Sweden; 2 Unit of Epidemiology, Institute of Environmental Medicine, Karolinska Institutet, Stockholm, Sweden; 3 Division of Cancer Studies, King’s College London, London, United Kingdom; 4 Department of Epidemiology and Biostatistics, Imperial College London, London, United Kingdom

**Keywords:** Clopidogrel, Proton-pump inhibitors, Gastrointestinal bleeding, Cardiovascular disease

## Abstract

**Background:**

It is unclear whether concomitant use of clopidogrel and proton-pump inhibitors (PPIs) increases the risk of recurrence of cardiovascular disease or death in patients at high risk of upper gastrointestinal (GI) bleeding.

**Methods:**

Based on the Swedish Patient Register, a cohort of cardiovascular disease (including acute myocardial infarction, stroke and angina, from 2006 to 2008) was selected from a population with any diagnosis of upper GI bleeding. Data on drug prescription was retrieved from the Prescribed Drug Register. Patients entered into the cohort after their first discharge for cardiovascular disease and were followed up to death, recurrence of cardiovascular disease, or 90 days. A Cox regression model was conducted and hazard ratios (HRs) with 95% confidence intervals (CIs) were estimated to evaluate the risks among users of different drug prescriptions.

**Results:**

Patients who were current users of only PPIs (HR 2.02, 95% CI 1.19-3.44), only clopidogrel (HR 1.14, 95% CI 0.53-2.45) and nonusers of both (HR 2.36, 95% CI 1.39-4.00) were at a higher risk of death compared with patients with a concomitant use. Results were similar among 1779 patients who had any history of upper GI bleeding (HR 2.05, 95% CI 1.18-3.54; HR 1.25, 95% CI 0.57-2.72; HR 2.30, 95% CI 1.33-3.98, respectively).

**Conclusion:**

Among patients at high risk of upper GI bleeding, those with a concomitant use of PPIs and clopidogrel were at a decreased risk of mortality, and possibly also a decreased risk of recurrence of cardiovascular disease.

## Background

Clopidogrel is a widely used medication for atherosclerotic diseases [1], particularly in the prevention of thrombotic events in stable cardiovascular disease, and as a secondary prevention after myocardial infarction and stroke [2,3]. Upper gastrointestinal (GI) bleeding is, however, a common and potentially serious adverse event in clopidogrel users [4]. Proton-pump inhibitors (PPIs), drugs that alleviate acid-related symptoms by inhibition of the hydrogen-potassium adenosine triphosphatase enzyme in gastric mucosa [5,6], are often prescribed to prevent such bleedings [5,7-9]. However, there are reports of an increased risk of mortality and recurrence of cardiovascular disease among patients with concomitant use of clopidogrel and PPIs [10-14]. A possible mechanism is that PPIs, which are mainly metabolized by the cytochrome system, might counteract the inhibitory effect of clopidogrel on platelet aggregation [15,16]. Nevertheless, some other studies have failed to find any interaction between clopidogrel and PPIs [17-19], or any increase in the risk of cardiovascular re-hospitalizations [20]. There is presently no general consensus on this issue, although the US Food and Drug Administration and the European Medicines Agency have stated that PPIs may reduce the effect of clopidogrel in the prevention of serious cardiovascular events [21,22].

During the past four years, accumulating relevant studies have been published. Few studies, however, have focused on patients with cardiovascular disease at high risk of upper GI bleeding [23]. Most have excluded patients with a history of GI bleeding [14,17,18,20,24] although these patients might be at high risk of a recurrence of severe GI bleeding. Furthermore, these studies have mainly evaluated the combined use of clopidogrel and aspirin together with PPIs which might dilute or mix the real interaction between clopidogrel and PPIs [25,26]. In addition, recurrence of cardiovascular disease is the major measured outcome in most previous studies rather than death. Death, though, is the most important and clearly measured outcome for cardiovascular disease and acute peptic ulcer bleeding. The recurrence of cardiovascular disease is, however, generally difficult to be identified completely.

In this cohort study, we focused on cardiovascular disease with any diagnosis of upper GI bleeding to clarify whether concomitant use of clopidogrel and PPIs increases the risk of recurrence of cardiovascular disease or death, compared with other treatment strategies.

## Methods

### Study design

**Figure 1:**
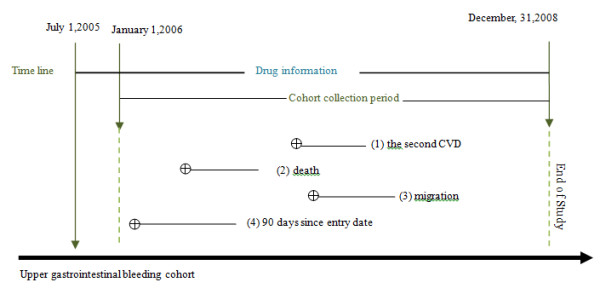
**The flow chart for study design.** The timeline showed how the cohort was defined. The Swedish Prescribed Drug Register started on July 1st, 2005. This study chose to start on January, 1st 2006 thus at least half-year drug exposure information can be guaranteed. Patients who discharged from hospitalizations for cardiovascular diseases since January, 1st 2006 were included in the cohort. They had been followed up the second cardiovascular event (1), death (2), migration (3), or end of study (4) within 90 days follow-up period. All of the patients had historical upper GI bleeding or they bled during/after the follow up period but before December, 31, 2008. Symbol ⊕ means diagnosis of cardiovascular diseases.

Within a population-based cohort of patients (1987 to 2008 in Sweden) with any hospitalization for complicated upper GI ulcer disease (International Classification of Disease 10th revision [ICD] diagnosis codes: K25-K28 with .0 .1 .2 .4 .5 .6), patients were selected for the final study if they had been hospitalized for cardiovascular disease from 2006 until the end of 2008. The entry date into the study cohort was the discharge date of the first cardiovascular disease hospitalization after January 1, 2006 (Figure 1). Patients were followed for 60, 90, 180 and 360 days to assess the outcomes death, re-hospitalization, or revisit to outpatient/primary health care for cardiovascular disease. In this study, we only present the results of the 90-day follow-up. The reason for this is that the short-term drug prescriptions after discharge for cardiovascular disease might more appropriately reflect the treatment in relation to fatality and recurrence. Moreover, restriction of the follow-up to only 60 days, 180 days and 360 days after entry rendered similar results to the 90-day follow-up (data not shown).

### Data sources

Data regarding basic patient information (admission date, discharge date, main diagnosis and secondary diagnosis) were retrieved from the *Swedish Patient Register*, which has been completed nationwide since 1987. Information on prescribed drugs and dates of dispatch were retrieved from the similarly complete Swedish Prescribed Drug Register (SPDR), which was initiated in July 1, 2005. This register contains information on age, sex, personal number, prescriber's profession and practice and, particularly, drug utilization and expenditure on prescribed drugs for the entire Swedish population. The defined cohort began on January 1, 2006, in order to ensure drug exposure covering at least half a year. The legally compulsory register of prescribed drugs in Sweden guarantees the complete capture of drugs exposure in this study. Death dates and underlying causes of death were identified from the nationwide complete *Swedish Cause of Death Register* (initiated in 1952), and data on cancer as a co-morbidity was collected from the nationwide complete *Swedish Cancer Register* (initiated in 1958).

### Identification of the study cohort

As we described above in the Study design section, patients with a first hospitalization for cardiovascular disease (including acute myocardial infarction, stroke, and angina) after January 1, 2006, were included in the study. These diseases were identified from the following ICD codes: main diagnosis or co-diagnosis of acute myocardial infarction (I21, I22), main diagnosis or co-diagnosis of ischemic stroke (I63, I64), and main diagnosis of angina (I20). In order to focus on clopidogrel and PPIs, we excluded patients with any filled prescription of aspirin (Anatomical Therapeutic Chemical [ATC] code: B01AC06, N02BA01, A01AD05) during the study period. Patients were also excluded from the final study cohort if they had previous acute myocardial infarction, stroke or angina hospitalizations within one year before entry, if they had emigrated before January 1, 2006, or if they had a cardiovascular re-hospitalization or had died less than 7 days after entry.

### Drug exposures

Current drug use was categorized into four groups: i) only PPIs, ii) only clopidogrel, iii) both clopidogrel and PPIs and, iv) no PPIs or clopidogrel. All the PPI types available in Sweden were included (omeprazole, pantoprazole, lansoprazole, rabeprazole, esomeprazole). The sample size did not allow separate analyses of single PPI groups. We calculated drug exposures at 30 days before the entry date as some patients might have had leftovers of previous PPIs or clopidogrel prescriptions at hand, which might have met their demands for current medications. We also analyzed the data using drug exposure that started from the entry date, or drug exposure 60 days before the entry. All of the results based on three definitions of exposures were similar. Thus, to make the study more concise, we only used the first definition of drug exposure. In Sweden, the typical prescription for PPIs or clopidogrel is for approximately 90 days or less. The extra 30 days plus 90 days of follow-up ensured enough time to cover any defined drug exposures. The ATC codes for clopidogrel were B01AC04 and B01AC30, and the ATC codes for PPIs were A02BC01-05 and A02BD01-06.

### Definition of outcomes

The outcomes under study were: recurrence of acute myocardial infarction (main or secondary diagnosis codes I21 or I22), stroke (main or secondary diagnosis codes I60-I64), angina (main diagnosis code I20), or all-cause mortality. We also specified hemorrhagic stroke and ischemic stroke from the total stroke patients.

### Co-morbidities

A co-morbidity score was calculated based on the following concomitant diagnoses: *i)* chronic heart failure (diagnosis code I50); *ii)* diabetes (diagnosis codes E10-E14); *iii*) chronic liver insufficiency (diagnosis codes K702-K704, K709, K717, K72, or K743-K746); *iv*) chronic renal insufficiency (diagnosis codes N18, N19, I132, I200, or I311); *v*) chronic obstructive lung disease (diagnosis codes J42-J44); *vi*) malignancy (diagnosis codes C00-C97). The reason for the selection of these diseases for the calculation of the co-morbidity score is because they are very commonly diagnosed and are composed of the most frequent causes of death in an elderly population [27,28]. The number of co-morbidities was categorized into four groups: no concomitant diagnosis, one concomitant diagnosis, two concomitant diagnoses, three or more concomitant diagnoses. History of cardiovascular disease was defined as any diagnosis of acute myocardial infarction, stroke, or angina at least one year before entry into the study cohort.

### Statistical analyses

A proportional risk model was conducted and hazard ratios (HRs) with 95% confidence intervals (CI) were estimated to evaluate the risk of death or cardiovascular disease in users of different categories of drug prescriptions. Current clopidogrel and PPI prescriptions were set as the reference. Patients who were diagnosed as having acute myocardial infarction at entry were analyzed as a subgroup and compared with patients diagnosed as having overall cardiovascular disease including acute myocardial infarction. Analyses were specified for patients who had a hospitalization for upper GI bleeding before or after the cardiovascular disease hospitalizations. The characteristics of patients for the diagnosis of upper GI bleeding were categorized into four groups based on a bleeding episode which happened before, during or after the study period. Group I contained patients who had a history of peptic ulcer bleeding before cohort entry. Group II contained patients who had a bleeding history and who bled again during the study period. Group III contained patients who had no history of bleeding but experienced a bleeding episode during the study period, and Group IV contained patients who did not bleed during but after the defined study period. Compared with the general population, all these patients are apt to have a higher risk of GI bleeding, therefore, reasonable subjects for analysis in a whole group [29,30]. Furthermore, we performed additional analysis for sub-groups. Since there are a limited number of patients in the third and fourth groups, we only did a sub-analysis for patients who had a history of upper GI bleeding (Groups I and II). All of the proportional models were adjusted for age (<65, 65–74, 75–84, ≥85), sex (male, female), history of cardiovascular disease (yes, no), history of bleeding (yes, no), and co-morbidity (0, 1, 2, 3 or more). To assess surveillance bias, additional analyses were conducted after excluding cases that died within 30 days after entry. SAS (the Statistical Analysis System, version 9.2, SAS Institute Inc., Cary, NC, USA) was used for all analyses. The study was approved by the Regional Ethical Review Board in Stockholm.

## Results

### Study participants

In total, 98 725 cases of peptic ulcer bleeding were ascertained from the Swedish Patient Register. Among them, 2285 first cardiovascular disease events were included in the study. Table 1 shows the baseline characteristics of the study participants at the time of inclusion. Among the patients with cardiovascular disease, we also did a sub-group analysis for acute myocardial infarction (Tables 1 and 2). The cohorts were followed for the outcomes death and recurrence of cardiovascular disease. Of the 2285 cardiovascular disease patients, 1536 (67%) were older than 75 years of age and 1269 (56%) were men. There were 812 (36%) current users of only PPIs, 165 (7%) current users of only clopidogrel, and 403 (18%) current users of concomitant prescriptions of PPIs and clopidogrel. In patients who only used one type of PPI, there were 878 omeprazole users, 162 esomeprazole users, 96 lansoprazole, 95 pantoprazole and 5 rabeprazole users. Among all participants, 245 (11%) died within the 90-day follow-up, 158 (7%) suffered ischemic stroke and 8 (0.3%) hemorrhagic stroke, while among 1817 (80%) patients with a diagnosis of upper GI bleeding before first entry into the cardiovascular disease cohort, 225 (13%) died within 90 days after discharge.

**Table 1:** Characteristics of study participants diagnosed with cardiovascular disease

**Characteristics**	**Death**				**Re-hospitalization for cardiovascular disease**			
	CVD* cohort		AMI** cohort		CVD* cohort		AMI** cohort	
	**Number (%)**		**Number (%)**		**Number (%)**		**Number (%)**	
***Total***	2219		673		2285		695	
*Men*	1227	55%	365	54%	1269	56%	374	54%
*Women*	992	45%	308	46%	1016	44%	321	46%
***Age group, years***								
*<65*	291	13%	57	8%	303	13%	62	9%
*65-75*	425	19%	95	14%	446	20%	98	14%
*75-85*	844	38%	280	42%	872	38%	292	42%
*> = 85*	659	30%	241	36%	664	29%	243	35%
***Upper gastrointestinal bleeding***								
I. Bleeding before entry	1730	78%	513	76%	1773	78%	527	76%
II. Bleeding before entry and during the follow up	49	2%	22	3%	44	2%	20	3%
III. New bleeding during the follow up period	86	4%	41	6%	71	3%	33	5%
IV. Bleeding after endpoints	354	16%	97	14%	397	17%	115	17%
***Cardiovascular disease history***								
*No*	1315	59%	456	68%	1361	60%	475	68%
*Yes*	904	41%	217	32%	924	40%	220	32%
***Comorbidities (number)***								
*0*	1382	62%	328	49%	1426	62%	340	49%
*1*	628	28%	244	36%	643	28%	250	36%
*2*	173	8%	81	12%	178	8%	85	12%
*≥3*	36	2%	20	3%	38	2%	20	3%
***Drug exposure***								
*Current clopidogrel and PPIs§*	309	14%	95	14%	403	18%	116	17%
*No current PPIs or clopidogrel*	861	39%	211	31%	905	40%	226	33%
*Current only PPIs*	847	38%	316	47%	812	36%	308	44%
*Current only clopidogrel*	202	9%	51	8%	165	7%	45	6%
***Patients with diagnosis of bleeding before entry***								
*Current clopidogrel and PPIs*	252	14%	78	15%	244	13%	77	14%
*No current PPIs or clopidogrel*	659	37%	154	29%	702	39%	164	30%
*Current only PPIs*	714	40%	265	50%	713	39%	266	49%
*Current only clopidogrel*	154	9%	38	7%	158	9%	40	7%
*subtotal*	1779		535		1817		547	

*CVD cardiovascular disease; **AMI acute myocardial infarction; §*PPIs* proton-pump inhibitors.

**Table 2:** Risk of death or recurrent cardiovascular events in 90 days follow-up among cardiovascular disease patients

**Drug exposures**	**Cardiovascular disease cohort**		**Acute myocardial infarction cohort**	
	**Death**	**Cardiovascular disease**	**Death**	**Cardiovascular disease**
	**§HR (95% CI)**	**§HR (95% CI)**	**§HR (95% CI )**	**§HR (95% CI)**
**Total cohort**				
Current clopodogrel and PPI** (reference)	1.00	1.00	1.00	1.00
*No PPI and no clopidogrel*	2.36 (1.39-4.00)	1.54 (1.05-2.24)	3.13 (1.47-6.68)	1.77 (0.92-3.41)
*Current only PPIs*	2.02 (1.19-3.44)	1.11 (0.75-1.65)	1.93 (0.91-4.11)	1.02 (0.52-1.99)
*Current only clopidogrel*	1.14 (0.53-2.45)	1.80 (1.15-2.83)	1.88 (0.70-5.03)	1.88 (0.85-4.08)
Patients with diagnosis of bleeding before entry*				
*Current clopodogrel and PPI(reference)*	1.00	1.00	1.00	1.00
*No PPI and no clopidogrel*	2.30 (1.33-3.98)	1.54 (0.98-2.40)	3.30 (1.47-7.41)	1.65 (0.78-3.47)
*Current only PPI*	2.05 (1.18-3.54)	1.04 (0.65-1.65)	2.12 (0.95-4.73)	0.80 (0.37-1.72)
*Current only clopidogrel*	1.25 (0.57-2.72)	1.84 (1.07-3.16)	2.26 (0.82-6.26)	1.78 (0.70-4.57)

*cohort members had history of upper gastrointestinal bleeding before entry into the cohorts. Results for patients who had any other hospitalizations for upper gastrointestinal bleeding were not shown due to the small number of cases.

**PPIs proton-pump inhibitors.

§*HR* harzard ratio; *CI* confidence interval. All of the proportional models were adjusted for age (<65, 65–74, 75–84, ≥85), sex (male, female), history of cardiovascular diseases (yes, no), history of bleeding (yes, no), and co-morbidity (0, 1, 2, 3 or more).

### Hazard ratios for different drug exposures in the cardiovascular disease cohort

The HR for risk of death within 90 days of follow-up was 2.02 (95% CI 1.19-3.44) for current users of only PPIs, 1.14 (95% CI 0.53-2.45) for current users of only clopidogrel, and 2.36 (95% CI 1.39-4.00) among patients with no PPI or clopidogrel prescription, compared with patients using PPIs and clopidogrel concomitantly (Table 2). Regarding the risk of recurrent cardiovascular disease, the corresponding HRs were: 1.11 (95% CI 0.75-1.65), 1.80 (95% CI 1.15-2.83), and 1.54 (95% CI 1.05-2.24), respectively.

### Hazard ratios for different drug exposures in the acute myocardial infarction cohort

In the acute myocardial infarction cohort, the HR for risk of death was 1.93 (95% 0.91-4.11) for current users of only PPIs, 1.88 (95% 0.70-5.03) for current users of only clopidogrel, and 3.13 (95% CI 1.47-6.68) for patients with no PPI or clopidogrel prescriptions. All the HRs for the risk of recurrent cardiovascular disease after acute myocardial infarction are not statistically different when compared to concomitant use of PPIs and clopidogrel.

### Patients with a diagnosis of upper GI bleeding before entry

Patients with any upper GI bleeding diagnosis before entry into the study were analyzed separately. The results were consistent with the main analyses (Table 2). An elevated risk of death (HR 2.05, 95% 1.18-3.54) was detected for current only PPIs, and an increased risk of recurrent cardiovascular disease (HR 1.84, 95% CI 1.07-3.16) was found for current use of only clopidogrel, compared with concomitant use of PPIs and clopidogrel. Restricting the cohort to those who had experienced a bleeding within one year before entry rendered similar results (data not shown).

## Discussion and conclusions

This study reveals no evidence of an increased risk of death or recurrence of cardiovascular disease among concomitant users of clopidogrel and PPIs in a population with a high risk of upper GI bleeding. Use of clopidogrel or PPIs alone seems to increase the risk of mortality and recurrent cardiovascular disease compared with concomitant use of these drugs.

Strengths of the study include the large sample size and the data collection from registers with complete nationwide coverage of the drug exposures and outcomes, i.e., recurrent cardiovascular disease and death. The nationwide register-based design counteracts selection and recall bias. Moreover, confounding was reduced by excluding patients with any recorded prescription of aspirin. There are, however, also several weaknesses. Filled prescriptions of PPIs and clopidogrel were regarded as the exposure, but there was no information on whether the patients had actually taken their medications. Also, PPI sub-types could not be analyzed separately due to the limited number, although the safety of pantoprazole has been specifically targeted in some studies [31]. More studies with a large sample size for possibly separating the analysis of PPI subtypes are warranted. In addition, it was not possible to entirely distinguish patients with a filled prescription after hospital admission from patients who still had drugs left from prescriptions filled before the index admission. Furthermore, we had no data on some important risk factors at baseline, such as tobacco smoking and alcohol consumption, which may bias the results [32]. We were, however, able to control for cardiovascular history and other co-morbidity as registered on hospital admission. Finally, confounding by indication could not be avoided completely in this observational study. Reasons for patients lacking any prescriptions might indicate that some completely recovered or that they might have had such severe symptoms that their physicians decided not to use any medication. These two opposite statuses of patients might make the confounding by indication less influential. The reason why some patients had only prescriptions of clopidogrel or only PPIs might be due to the recent reports of the increased risk of concomitant use of these two drugs rather than any specific disease indications. On the other hand, the consistently increased risk of recurrence of cardiovascular disease in clopidogrel only patients compared with the concomitant users may indicate the competing risk of death in this group.

Whether concomitant prescriptions of clopidogrel PPIs are associated with an increased recurrence of cardiovascular events is controversial [33]. Some studies have indicated a concern that the interaction between clopidogrel and PPIs could impair the treatment effect of clopidogrel [10-14,16], but others have not found any association. Instead, an independent cardiovascular risk by PPIs alone has been detected in a few studies. Several relevant meta-analyses have been published since 2010. Hulot and his coworkers initiated a meta-analysis in 2009 focusing on clopidogrel-treated patients with cytochrome P450 2C19*2 loss-of-function allele or the co-administration of PPIs. The results displayed an increased risk of major adverse cardiovascular events in the co-administration of clopidogrel and PPIs [34]. During the same year, the study by Kwok et al. showed that propensity-matched or randomized trial participants had no associated cardiovascular risk with PPIs, whereas other observational studies generally showed a significant association [35]. The same team published an updated study in 2012 with a focus on the potential risk with individual PPIs. They found an elevated risk of PPI therapy alone is associated with an adverse cardiovascular risk. They also admitted that major adverse cardiovascular events were mostly limited by the moderate to substantial heterogeneity of the studies included for meta-analysis. The high possibility of confounding and bias was indicated [31]. Siller-Matula and his colleagues published another meta-analysis in 2010. Their results indicated that concomitant PPI use might be associated with an increased risk of cardiovascular events but does not influence the risk of death. Articles included in these meta-analyses were not completely the same due to different inclusion criteria and time of publication. They further confirmed the presence of significant heterogeneity which might indicate that the evidence is biased, confounded, or inconsistent [36]. The clinical validity or relevance of the hypothesized PPI-clopidogrel interaction, thus, remains questionable.

The present study indicates that the co-administration of clopidogrel and PPIs results in a better effect regarding the prevention of death in patients with a high risk of GI bleeding. The current findings are consistent with some previous studies [17-20,37-40]. For example, a cohort study in the US and Canada which included 18 565 patients with acute coronary syndrome undergoing percutaneous coronary intervention (PCI) found no conclusive evidence of a clopidogrel-PPI interaction, even though there was a slightly increased risk of hospitalization for myocardial infarction. A study from Austria included 300 patients with coronary artery disease who had PCI. The platelet reactivity index was measured in patients with and without PPIs, and no interaction was found between clopidogrel and pantoprazole or esomeprazole [17]. One big cohort study from Denmark found that the apparent association between recurrent myocardial infarction and the use of PPIs with clopidogrel is affected by confounding by indication. The association is not present when confounding is addressed by design [41]. Although indications of reduced antiplatelet activity have been found *in vivo* in the case of PPI administration in clopidogrel users, the interaction between antiplatelet agents and PPIs at the enzymatic level does not seem to result in worse clinical outcomes.

In summary, recent studies have shown conflicting and inconsistent data regarding the adverse interaction of clopidogrel and PPIs. Together with our study, we think clinicians should focus on the potential harm from ulcers or hemorrhage before deciding to omit PPIs in patients taking clopidogrel. In selected patients at high-risk of GI bleeding, clinicians should in fact be very cautious and cannot overlook the life-threatening risk that bleedings represent.

## Competing interests

All authors have no competing interests to declare: no support from any organization for the submitted work; no financial relationships with any organizations that might have an interest in the submitted work in the previous three years and no other relationships or activities that could appear to have influenced the submitted work.

## Authors’ contributions

QW participated in the study design, carried out the statistical analysis and drafted the manuscript. RL collected the data, contributed to data arrangement, data analysis, and interpretation of results and helped to draft the manuscript; JL participated in the study design, contributed to interpretation of the results and drafted the manuscript; LY conceived of the study, contributed to the acquisition of the data, participated in the study design and helped to draft the manuscript. All authors read and approved the final manuscript.

## Pre-publication history

The pre-publication history for this paper can be accessed here:

http://www.biomedcentral.com/2050-6511/15/22/prepub
